# Fast neutron mutagenesis in soybean enriches for small indels and creates frameshift mutations

**DOI:** 10.1093/g3journal/jkab431

**Published:** 2021-12-15

**Authors:** Skylar R Wyant, M Fernanda Rodriguez, Corey K Carter, Wayne A Parrott, Scott A Jackson, Robert M Stupar, Peter L Morrell

**Affiliations:** 1 Department of Ecology and Evolutionary Biology, University of California, Irvine, CA 92697, USA; 2 Department of Agronomy and Plant Genetics, University of Minnesota, St. Paul, MN 55108, USA; 3 Department of Crop and Soil Sciences, University of Georgia, Athens, GA 30602, USA

**Keywords:** mutation, mutagen, fast neutron, soybean, single-nucleotide variants

## Abstract

The mutagenic effects of ionizing radiation have been used for decades to create novel variants in experimental populations. Fast neutron (FN) bombardment as a mutagen has been especially widespread in plants, with extensive reports describing the induction of large structural variants, *i.e.*, deletions, insertions, inversions, and translocations. However, the full spectrum of FN-induced mutations is poorly understood. We contrast small insertions and deletions (indels) observed in 27 soybean lines subject to FN irradiation with the standing indels identified in 107 diverse soybean lines. We use the same populations to contrast the nature and context (bases flanking a nucleotide change) of single-nucleotide variants. The accumulation of new single-nucleotide changes in FN lines is marginally higher than expected based on spontaneous mutation. In FN-treated lines and in standing variation, C→T transitions and the corresponding reverse complement G→A transitions are the most abundant and occur most frequently in a CpG local context. These data indicate that most SNPs identified in FN lines are likely derived from spontaneous de novo processes in generations following mutagenesis rather than from the FN irradiation mutagen. However, small indels in FN lines differ from standing variants. Short insertions, from 1 to 6 bp, are less abundant than in standing variation. Short deletions are more abundant and prone to induce frameshift mutations that should disrupt the structure and function of encoded proteins. These findings indicate that FN irradiation generates numerous small indels, increasing the abundance of loss-of-function mutations that impact single genes.

Statement of significance

While irradiation mutagenesis is commonly viewed as a method to induce large structural variants in genomes, we find enrichment for small insertion and deletion (indel) variants. Induced mutations are likely to constitute a relatively small portion of the genetic variation present in crop species; irradiation mutagenesis is useful for altering genomes by introducing small indels into single genes or disrupting gene clusters by creating structural variants.

## Introduction

Naturally occurring mutations have long been recognized as the primary source of genetic variation used for selection in plant breeding programs. Mutations induced by irradiation and mutagenic chemicals have also been important for generating variation when naturally occurring genetic variation for a trait was absent or insufficient (Spencer-Lopes *et al.* 2018). The advent of CRISPR and other site-directed nucleases has enabled targeted nucleotide changes, thus eliminating much of the randomness from the generation of de novo variation. Nevertheless, not all genomic sites lend themselves to editing for reasons that are still not clear (Naim *et al.* 2020).

The advent of genetic engineering technology for crops cast mutations in a new light. The US Food and Drug Administration initially speculated that unintended mutations caused by the insertion of a transgene or mutations occurring during the tissue culture portion of the transformation process could trigger the production of unknown toxins, negatively affecting human food and animal feed safety (Kessler *et al.* 1992). As a result, an extensive and expensive system for testing the safety of genetically modified crops has been instituted (Kuiper *et al.* 2001; Cellini *et al.* 2004). It was also hypothesized that these mutations could cause crops to have undesirable environmental effects (Latham *et al.* 2006; Wilson *et al.* 2006). More recently, the potential for CRISPR and other site-directed nucleases to induce genetic changes beyond targeted nucleotide sites (*i.e.*, “off-targets”) has created renewed interest in genome-level screens for de novo mutations. This is partly due to lingering concerns over the safety implications of these off-target effects (Wolt *et al.* 2016; Graham *et al.* 2020). The best way to assess the safety implications of off-target edits is by comparison to natural and induced mutations that have a history of safe use in breeding programs.

Comparing naturally occurring variants to variants in mutagenized lines provides a best-case scenario for determining how and if induced mutations differ from naturally occurring variants. Mutagenized plants with heritable phenotypic differences from the original experimental line must carry mutations capable of altering the phenotype. Mutagenesis experiments use a mutagen exposure at a level that would be lethal to a proportion of treated individuals. Standard measures of toxicity use an “LD_50_” (median lethal dose), meaning that half of all treated individuals do not survive the mutagenic treatment at the level of application. Mutagenized individuals that survive are likely to have been subject to mutation but carry induced genetic changes that are less damaging.

While the mutagenic effects of FN radiation have been successfully applied to create genetic novelty in plants (Kumawat *et al.* 2019), the full spectrum of FN-induced mutations is poorly understood. FN bombardment can create large structural variants, including deletions, duplications, inversions, and translocations, but single-nucleotide point mutations and small indels have also been reported (Belfield *et al.* 2012). Previous studies have characterized large deletions and duplications in soybean (Bolon *et al.* 2014; Anderson *et al.* 2016). In *Arabidopsis thaliana*, there was enrichment for single-base substitutions, primarily at pyrimidine dinucleotides (Belfield *et al.* 2012). In rice, single-base substitutions constituted the most abundant mutation type, but deletions mutated the largest number of genes (Li *et al.* 2016).

Distinct patterns of mutation tend to predominate for both spontaneous and mutagen-induced changes. For spontaneous mutations, transitions (changes between pyrimidines or between purines) are observed at a higher relative rate than transversions (changes between pyrimidines and purines; Fitch 1967). New mutations can be quite distinct from standing variation in populations, a phenomenon explored in deep-resequencing panels (Schaibley *et al.* 2013). Short insertions and deletions, typically smaller than a sequence read length, are also readily detectable with Illumina short-read sequencing (Gilad *et al.* 2009). Short deletions relative to a reference genome are potentially the most readily detectable indel events, owing to fewer issues with initial read mapping. Short indels may be particularly abundant in low complexity sequence, including mononucleotide repeats. Low complexity sequence also provides challenges due to limitations of sequencing chemistry and alignment algorithms.

Mutation rates vary across the genome, though the causes of this variation are not completely understood (Hodgkinson and Eyre-Walker 2011). Chromosomal-scale patterns in mutation rate are impacted by factors such as GC content (Webster *et al.* 2003; Schaibley *et al.* 2013), local recombination rates (Schaibley *et al.* 2013), and methylation rates (Takuno and Gaut 2013). However, the immediate context of variants appears to have the greatest impact on the nature of variants observed (Aggarwala and Voight 2016; see also Morton 2003; Morton *et al.* 2006). The most abundant mutation observed in many organisms is the C→T transition, frequently in a CpG context (Nachman and Crowell 2000; Hwang and Green 2004). That is, a cytosine is bound by a phosphate bond to guanine on the same side of the nucleotide strand (or nucleoside) and in the 5ʹ–3ʹ orientation. As a shorthand for mutations that could have arisen on either strand, we use C→T*, which refers both to C→T changes and the reverse complement G→A. These changes cannot typically be distinguished in resequencing studies. The effect of context varies based on mutagenizing factors. For example, when used in plants, the mutagen ethyl methanesulfonate tends to induce transitions in a GC-rich context (Henry *et al.* 2014). These results suggest that observed mutations and the context in which they occur are mutagen dependent and based on specific biochemistry. The specific context in which mutations are more likely to occur matters because the immediate flanking nucleotides can make certain classes of large-effect mutations, such as stop codons, less probable.

Quantification of the influence of local nucleotide sequence context on mutation requires that mutations are divided into specific classes. Identification of the mutational context of insertions and deletions is often not directly observable because multiple equiprobable local nucleotide sequence alignments are possible around the mutation (Clegg and Morrell 2004a, 2004b). There are 12 possible single-nucleotide mutations. If the local context affects the potential occurrence of these variants, particular nucleotides would be overrepresented among the immediately flanking sequences. Ideally, an approach would also account for the probability of sampling each of the nucleotides rather than relying on an equiprobable occurrence of all four nucleotides. It should also permit the examination of the first- or second-order interactions in terms of the presence of particular flanking nucleotides (Zhu *et al.* 2017).

Here, we investigate the differing nature of induced *vs* standing variants among panels of different soybean genotypes. This includes 27 soybean lines subject to FN mutagenesis compared to standing variation in 107 diverse soybeans. Because the potential for creating large structural changes is well documented (Bolon *et al.* 2014), we focus on single-nucleotide and small-indel variants that can be readily detected with short sequence reads (Albers *et al.* 2011). We start by determining if there is a difference in the mutational spectrum of induced mutations relative to standing variation. We then investigate if local context (flanking nucleotides) affects the frequency and type of mutations.

## Materials and methods

### Resequencing data

To examine the effects of fast neutron (FN) mutagenesis on single-nucleotide variation in soybean, we used published resequencing data sets that included FN-treated lines (Bolon *et al.* 2011, 2014) and untreated lines that serve as a control (Valliyodan *et al.* 2016). Resequencing data from 30 lines subject to FN mutagenesis, all from the M92-220 line (Bolon *et al.* 2014), were the treatment group. We also reanalyzed the sequence from three individuals from the M92-220 line that were nonmutagenized. Soybean lines analyzed here were exposed to 8, 16, or 32 Gy of radiation and four to eight generations of self-fertilization (Table 1). Three of the 30 mutagenized lines were removed from analysis due to contamination detected during the de novo variant analysis. Of the remaining 27 lines, two sets of three siblings were derived from the same mutagenized plant (Table 1). None of the lines exposed to mutagenesis displayed aberrant phenotypes. We used published resequencing data from 106 soybean lines, including wild, landrace, and elite accessions reported by Valliyodan *et al.* (2016) and a single accession of the cultivated line M92-220 reported by Bolon *et al.* (2011) to identify naturally occurring variants in soybean.

**Table 1:** Sample information from the fast neutron lines and genetic background line (M92-220.x1.04.WT) from Bolon *et al*. (2014), excluding three lines that were discarded due to contamination

Sample name	Generations of selfing	Fast neutron dosage (Gy)	Mean depth	Total indels	Total SNPs
M92-220.x1.04.WT	N/A	N/A	63	298,229	1,369,820
2012BM7F223	7	8	28	25	56
2012CM7F040P05*	7	16	30	38	76
2012CM7F040P06*	7	16	40	47	72
2012CM7F040P07*	7	16	29	9	23
2012CM8F030P02+	8	16	28	27	39
2012CM8F030P07+	8	16	30	18	20
2012CM8F030P09+	8	16	29	18	35
2012DM8F016P02	8	16	30	51	89
4R30C22acr626MN13	6	16	20	34	41
5R39C03Dr334cMN12	3	32	20	14	8
FN0112228.06.02.01.M5	5	16	28	53	62
FN0112885.02.06.03.M5	5	16	33	32	49
FN0131633.06.01.M4	4	16	29	33	56
FN0163764.04.01.M4	4	32	19	36	63
FN0164160.03.02.01.01.M6	6	32	28	50	87
FN0164472.x3.06.01.M5	5	32	29	49	83
FN0170228.07.35.01.M5	5	16	25	38	64
FN0170712.06.41.01.M5	5	16	29	41	80
FN0171501.01.02.M4	4	32	31	38	67
FN0172932.09.08.01.M5	5	16	24	15	16
FN0173217.03.09.01.M5	5	16	30	25	56
FN0175143.05.06.01.M5	5	16	32	34	45
FN0175501.x2.02.01.M5	5	16	29	29	50
FN0190069.01.01.M4	4	32	26	31	70
R18C55Dhaar437MN13	6	32	20	23	25
R52C55Dadr564MN13	5	32	18	20	22
RP8DM5r597MN13	6	32	19	41	79

The “*” identifies three samples that are siblings (family 1). The “+” identifies three samples that are siblings (family 2).

An important step in the identification of de novo variants is the identifications of differences between the treated line and the reference genome and also heterogeneity and heterozygosity in the treatment line. We made use of previous Illumina paired-end resequencing of the original variety used for mutagenesis, M92-220. This included 100-bp Illumina sequencing from Bolon *et al.* (2011). We also collected additional whole-genome sequencing data from two different plants from the same seed lot, as there is known to be heterogeneity within the M92-220 seed stock (Bolon *et al.* 2011). These newly collected sequences have been deposited in the NCBI SRA database, project number PRJNA670564. Reference-based read mapping is as described below, except for the 10× Genomics samples, which made use of the Long Ranger software version 2.2.2 https://support.10xgenomics.com/genome-exome/software/downloads/latest (Accessed: 2021 December 21). The Long Ranger software was also used to identify structural differences between a reference genome and a query sample that are supported by both linked reads and split read information.

### Read alignment and variant calling

Read alignment and variant calling were implemented using publicly available software integrated with bash scripts in the “sequence_handling” workflow (Hoffman *et al.* 2018). Configuration files and scripts used for analysis are available at https://github.com/MorrellLAB/Context_Variants_Soy (Accessed: 2021 December 21). Reads were aligned to soybean reference genome “Gmax_275_v2.0,” a part of the Phytozome 11 release (https://jgi.doe.gov/data-and-tools/phytozome/; Accessed: 2021 December 21). Alignment parameters were adjusted to account for the levels of nucleotide diversity in soybean reported by Hyten *et al.* (2006).

The variant call format (VCF; Danecek *et al.* 2011) files for the 27 FN lines, 107 diverse lines, and four M92-220 lines were filtered with bcftools (Li 2011) to include only variants and sample genotypes that satisfied a set of quality criteria. Filtering criteria are implemented in a shell script in the project repository https://github.com/MorrellLAB/Context_Variants_Soy (Accessed: 2021 December 21). Specifically, we filtered standing variants into two classes based on nonreference allele count. The common class contained all variants with an allele count of three or higher, and the rare class contained all variants with an allele count of two or less. VCF and related files are available through a Data Repository of University of Minnesota (DRUM) archive at https://doi.org/10.13020/0s9b-p605 (Accessed: 2021 December 21).

Descriptive statistics from VCF files, including observed heterozygosity and average pairwise diversity, were calculated using scikit-allel (Miles *et al.* 2019). The transition/transversion ratio, nature of variants relative to the reference (mutational spectrum), and SNP quality scores were calculated using bcftools (Li 2011).

### Identifying de novo variants

**Figure 1:**
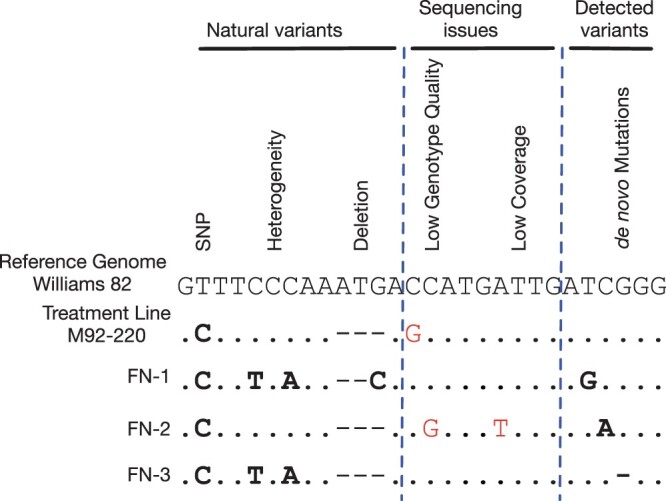
The detection of de novo mutations in FN treatment lines requires the filtering of naturally occurring variants present in the initial treatment lines, including deletions in the treatment line where reference-based read mapping cannot identify variants. Sequencing issues, including low-quality variant calls and low-coverage regions, are removed to isolate variants unique to treatment lines. Accurate base calls are shown in bold, even if they are filtered out. Erroneous base calls are shown in red.

A major challenge with the identification of de novo variants is distinguishing variants present in a line prior to modification from de novo mutations. In practice, this involves filtering out several classes of variants, including (1) differences between the genotype used for mutagenesis and the reference genome, (2) regions of the genome where variants cannot be identified, such as deletions in the mutagenized germplasm (that are a natural part of standing variation) relative to the reference, and (3) heterozygosity within lines or heterogeneity among lines of the mutagenized germplasm (Anderson *et al.* 2016; Michno and Stupar 2018; Figure 1). It is also necessary to account for variants that arise during seed maintenance prior to mutagenesis. Even if all lines are derived through single seed descent, a large seed increase over multiple generations is necessary to create the bulk seed subject to FN treatment. To account for this, we add 10 generations of mutation to estimates of variation in the M92-220 seed lot before FN treatment.

Nucleotide sequence diversity in the FN panel was estimated using pylibseq, a Python interface for the libsequence library for evolutionary genetic analysis (Thornton 2003). All high-confidence SNPs in FN lines are homozygous singletons. We estimate θπ (Tajima 1983) using a haploid sample size of 27 for the inbred lines.

### Variant flanking sequencing

To identify the most frequent motifs associated with each class of observed single-nucleotide changes, we used the Mutation Motif Python package (Zhu *et al.* 2017). The program performs a comparative statistical analysis of the nucleotide state of positions that immediately flank observed nucleotide variants. The largest effects tend to occur at positions 1 or 2 bp up and downstream of variants (Zhu *et al.* 2017; see Supplementary Figure S1). Mutation Motif identifies significant differences in composition at variant sites by drawing a random sample of flanking positions from the immediate context while controlling for positional effects by focusing on a window of sequence around each variant.

We developed a script called SNP_Context (in Bash and Python) that draws samples of variant-adjacent sequence from a reference genome (https://github.com/carte731/SNP_Context; Accessed: 2021 December 21). The script reads a VCF file, confirms variant states relative to the reference, checks for overlaps among variant positions, and creates a sequence FASTA file compatible with Mutation Motif (Zhu *et al.* 2017).

Variant annotation was performed using Variant Effect Predictor (VeP; McLaren *et al.* 2016) with gene models provided by Schmutz *et al.* (2010). VeP reports the total number of transcript changes induced by a variant (Table 2).

**Table 2:** The number and proportion of affected transcripts in functional classes annotated by VeP

Consequence (variant) type	FN27	Proportion (%)	Natural—common	Proportion (%)	Natural—rare	Proportion (%)
splice_donor	0	0.00	1,786	0.01	939	0.02
splice_acceptor	5	0.16	1,838	0.01	922	0.02
stop_gained	0	0.00	4,539	0.03	2,797	0.05
Frameshift	34	1.11	11,359	0.07	6,364	0.11
stop_lost	0	0.00	819	0.01	235	0.00
start_lost	0	0.00	810	0.01	317	0.01
inframe_insertion	0	0.00	4,959	0.03	1,665	0.03
inframe_deletion	0	0.00	4,603	0.03	2,490	0.04
protein_altering	0	0.00	242	0.00	127	0.00
Missense	68	2.21	204,987	1.28	87,537	1.54
splice_region	13	0.42	38,143	0.24	14,205	0.25
start_retained	0	0.00	112	0.00	28	0.00
Synonymous	29	0.94	167,608	1.04	58,830	1.03
stop_retained	0	0.00	436	0.00	148	0.00
coding_sequence	0	0.00	486	0.00	256	0.00
5′ UTR	19	0.62	140,683	0.88	48,300	0.85
3′ UTR	28	0.91	213,299	1.33	73,596	1.29
Intron	263	8.57	1,789,836	11.16	639,990	11.25
Upstream	905	29.48	5,325,487	33.20	1,721,428	30.25
Downstream	820	26.71	4,764,026	29.70	1,572,140	27.63
Intergenic	886	28.86	3,364,380	20.97	1,458,227	25.63
Total	3,070	100	16,040,438	100	5,690,541	100

The fast neutron treatment panel has a larger proportion of transcripts annotated as containing a frameshift or missense variant than the common or rare natural variation panels.

To determine the total number of sites in the genome that include the most likely sequence motif for FN mutations, we searched all possible 2-, 3-, 4-mers constituting the most frequent sequence context. We examined sequence context for the two most abundant classes of de novo FN variants. This analysis made use of the EMBOSS (Rice *et al.* 2000) “compseq” tool.

## Results

### Identification of standing and de novo variants

To examine the effects of FN mutagenesis on single-nucleotide variation in soybean, we made use of two published resequencing data sets (Bolon *et al.* 2011, 2014) that included 27 soybean lines subject to 8, 16, or 32 gray units (Gy) of FN radiation (Table 1, Supplementary Figure S2). Resequencing data for this accession had a mean mapped read coverage of 27.2× (±5.11; Table 1). Coverage relative to the number of SNPs and indels identified per line is shown in Supplementary Figure S3. We also examined one nonmutagenized soybean inbred line (M92-220) sequenced to 63× that served as the initial parental line for the FN mutant population (Supplementary Figure S4). Sequenced accessions were subject to four to eight generations of self-fertilization before sequencing (Table 1).

Our goal was to identify induced mutations in the M92-220 treatment lines subject to FN exposure. A necessary first step was the identification of variants that differentiate M92-220 from the “Williams 82” reference genome (Schmutz *et al.* 2010; Figure 1). To identify these variants, we included resequencing from prior studies (Bolon *et al.* 2011, 2014), along with newly collected Illumina resequencing from M92-220 with and without 10× Genomics linked reads. To provide a contrast between induced mutations found in treatment lines and natural variation, we made use of a published resequencing dataset of 106 soybean lines reported by Valliyodan *et al.*, (2016; Supplementary Figure S4). The 106 line dataset (Valliyodan *et al.* 2016) was also used to filter naturally occurring variants from our FN-treated lines.

Reference-based read mapping from three individuals of the M92-220 inbred line identified a total of 1,668,049 single-nucleotide variants with an observed transition/transversion ratio (Ts/Tv) of 1.90. We also identified 299,980 sequence insertions or deletions (indels) smaller than 280 bp) which are differences from the Williams 82 reference. The 10× Genomics-linked reads provided improved potential to detect intermediate-length structural variants. Using a single accession of M92-220 sequenced with 10× Genomics-linked reads, we identified 495 additional indels that were not identified by paired-end sequencing alone, ranging in size from 17,767 to 499,355 bp. Indels include 105 deletions relative to the reference, 147 duplications, 16 inversions, 10 translocations, and 217 variants with types that could not be identified. Deletions in M92-220 relative to the Williams 82 reference constituted 3.82% of the total genome size. De novo variants could not be detected in regions that harbored deletions relative to the reference, so these regions were masked from further analysis.

Standing variation, in terms of reference-based read mapping of the 106 samples from Valliyodan *et al.* (2016) was assessed in samples with mean mapped read coverage of 13.96× (±1.33) and M92-220 from Bolon *et al.* (2011, 2014) with 63× coverage. We identified 9.7 million SNPs with an observed Ts/Tv = 1.94 and 1.5 million indels, for a total of 11,196,099 variants. Estimated pairwise diversity (Tajima 1983), reported as *θ*_π_ = 4 N_e_µ at all SNP sites is shown for chromosome 1 in Supplementary Figure S5. Genome-wide diversity was *θ*_π_ ∼2 × 10^−3^ in the full panel from Valliyodan *et al.* (2016), which included soybean cultivars, landraces, and wild accessions that represent the variation present in the modern varieties or that is accessible to breeders.

Standing variants were partitioned into two classes based on frequency to allow for a more direct comparison with FN variants (Supplementary Figure S6). The first class included common variants with a nonreference allele count of at least three in our dataset. The second class included rare variants with a nonreference allele identified in either one or two genotypes. These rare variants have experienced fewer generations of selection and are likely more similar in terms of mutational spectrum and context to variants produced by spontaneous mutational processes.

### Variation in FN lines

FN lines exhibited a total of 2296 de novo variants in the homozygous state, including indels, for a mean of 85.3 (±34.0) new mutations per line. This total included variants distributed relatively evenly across all 20 soybean chromosomes and 24 of the 1170 scaffolds in the Gmax_275_v2.0 genome assembly (Schmutz *et al.* 2010). We identified 1430 SNPs, which is an average of 53.0 (±23.0) per line. We also identified 866 indels for an average of 32.1 (±11.9) per line. Among these variants, those most likely to disrupt genes include 34 frameshift and 68 missense changes that impact an average of 2.15 (±1.76) genes per plant.

We used observed variants across the 966-Mb reference genome in a haploid sample to estimate SNP-level diversity. This results in a pairwise diversity estimate of *θ*_π_ = 1.1 × 10^−7^ for de novo mutations (Supplementary Figure S5B). Note that *θ*_π_ estimates are parametric and not dependent on sample size (Tajima 1983). Thus, the very low polymorphism observed in Supplementary Figure S5B results from de novo mutations either from the FN treatment or due to spontaneous mutations arising during line maintenance.

**Figure 2:**
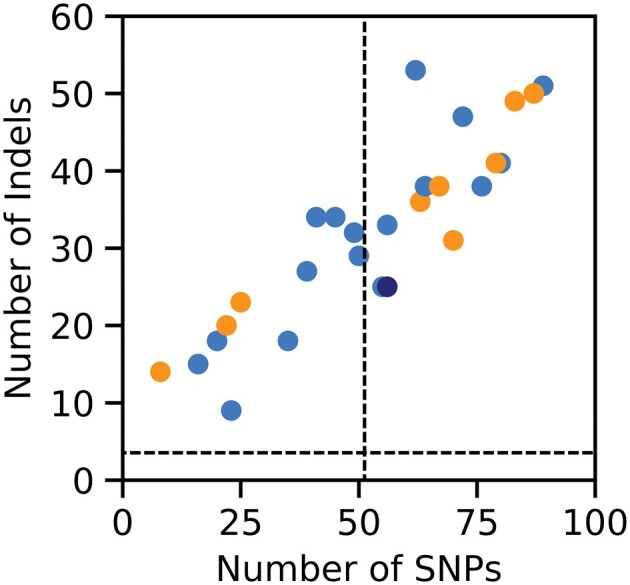
The number of variants identified in each fast neutron line. The samples were exposed to 8 Gy (dark blue), 16 Gy (light blue), or 32 Gy (orange). Dotted lines indicate the expected number of SNPs and indels based on average mutation rates.

Within individual lines, mutations can be identified as SNPs and indels. The number of SNPs and indels observed in a line is correlated (*r*^2^ = 0.778) and significant in a paired *t*-test (*P* = 3.18e−08). There is a relatively large variance in mutation number, with the lowest number of observed variants occurring in an FN line with the highest (32 Gy) exposure (Figure 2). Another factor that could impact observed diversity is the number of generations of maintenance of each line. After mutagenesis, lines were subject to four to eight generations of self-fertilization (Table 1). Using a two-way ANOVA, neither the dosage of radiation (in Gray units) nor generations of selfing have a significant effect on the number of observed SNPs or indels in individual lines (Figure 2). The limited predictive value of FN dosage or selfing generations likely reflects the limited number of observations (new mutations) as part of a stochastic mutation process.

We can estimate the expected number of mutations in the FN lines in the absence of the single-generation FN treatment based on average per generation mutation rates. A more difficult factor to account for is the within-line heterogeneity present within the seed stock of M92-220 used for initial mutagenesis (Stec *et al.* 2013). For this analysis, we estimated that 10 generations of self-fertilization (accompanied by lineage-independent spontaneous mutations) occurred before the mutagenesis treatment. We make use of the mutation rate estimates of Ossowski *et al.* (2010) from a mutation accumulation experiment in *A.thaliana*. Using the mean (6.53 × 10^−9^) of nucleotide substitution rates estimated by Ossowski *et al.* (2010), we expect a mean of 50.57 SNPs per line. Using a mean indel mutation rate estimate of 0.47 × 10^−9^ (Ossowski *et al.* 2010), we expect a mean of 3.08 indels per line (Figure 2). The observed number of SNPs per line is very close to the expectations based only on spontaneous mutations. The observed number of indels per line is more than an order of magnitude greater than expected based on spontaneous variants over generations.

### Mutational spectrum

**Figure 3:**
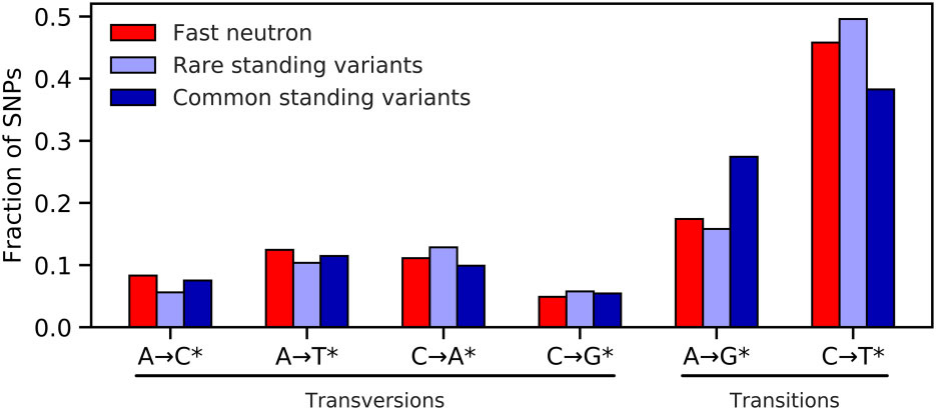
The mutational spectrum of fast neutron variants, rare standing variants (nonreference allele count of two or fewer), and common standing variants (nonreference allele count of three or higher). Each class combines the displayed change and its reverse complement [*e.g.*, the notation C→T* indicates that both cytosine to thymine and the reverse complement guanine to adenine mutations are binned into a single class (see text)].

The SNP mutational spectrum is similar for the 107 diverse lines and the 27 FN lines (Figure 3). Transitions are more common than transversions, with C→T* transitions being particularly abundant (Figure 3). Among the 107 lines, the second most abundant class of mutations is A→G* transitions (Figure 3), with common standing variants particularly enriched for this class. For FN lines, C→T* transitions are slightly more abundant than in the 107 lines in the standing variation panel, while A→G* transitions are slightly less abundant, as described below.

### Context of mutations

The survey of standing variation in the 107 lines identified 9,708,345 SNPs. The most abundant class of variants includes 4,035,491 (41.57%) C→T* transitions, and the least abundant includes 532,675 C→G* (5.49%) transversions. The very large sample size for standing variation provides the potential to observe all four nucleotides at each of the four flanking nucleotide positions. In standing variation, C→T mutations tend to be followed by G (guanine) at either position 1 or position 2 following the SNP (see Supplementary Figure S1 for explanation). While the CG motif provides the context for the most abundant class of mutations, it is the rarest of all two-nucleotide motifs in the soybean genome, constituting only 1.6% of all two-nucleotide combinations. In contrast, the AA and TT motifs make up 11.9% of all two nucleotide combinations.

**Figure 4:**
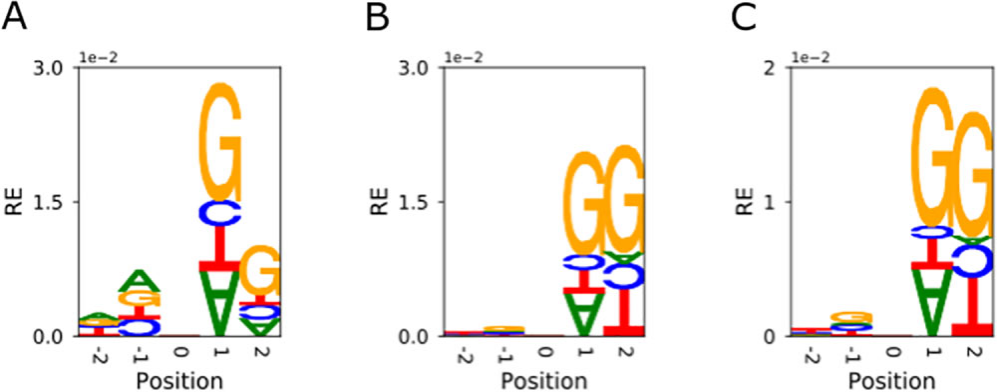
The nucleotide sequence context in which C→T transitions occur relative to the reference genome for fast neutron variants (A, *N* = 319), rare standing variants (B, *N* = 647,614), and common standing variants (C, *N* = 1,350,269). The relative height of a letter indicates its relative entropy (RE), with overrepresented and underrepresented bases portrayed as right side up and upside down, respectively. The null expectation (zero RE) is based on a nearby nucleotide of the same base that has mutated, chosen at random.

The contextual pattern for C→T transitions in the FN lines is similar to those of the diverse panel. However, comparison in de novo variants is limited by the number of observations, with C→T* constituting 655 of the 1430 (45.8%) observed de novo variants. C→T changes in the FN lines were also found to be followed by G at positions 1 or 2, but the relative effect of position 1 appears to be larger (Figure 4). The −1 position was typically A or G, but this was a smaller effect. However, the number of these combinations was limited by sample size.

#### Indel variation

**Figure 5:**
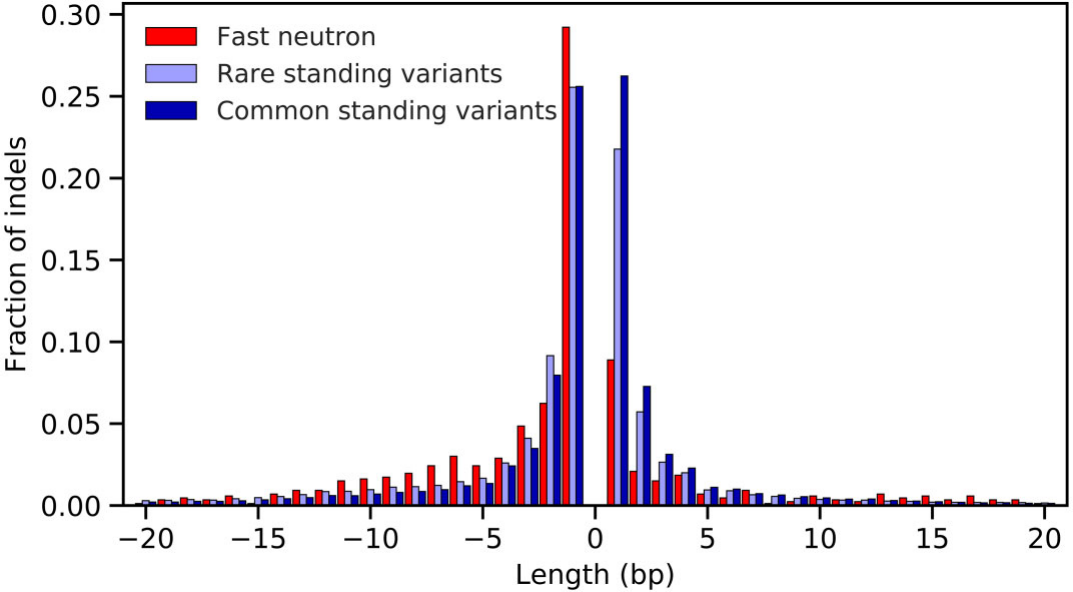
The distribution of insertion and deletion lengths for fast neutron variants, rare standing variants (nonreference allele count of two or fewer), and common standing variants (nonreference allele count of three or higher). Insertions are shown as positive values and deletions as negative values. Variants with lengths greater than 20 bp are not shown.

The distribution of indel sizes differed between variants observed in the FN, rare standing, and common standing classes (Figure 5). In FN lines, there are many more deletions than insertions, with only 28.5% of indels as insertions. In standing variation, the two classes are more evenly represented, with 860,649 deletions and 756,095 insertions (46.8% insertions). Rare standing variants should be impacted less by purifying selection than the common standing variants and thus are most similar to expectations for new mutations. Comparing the rarest indels in the standing variation to the same size class in the FN lines using a paired *t*-test identified significant differences for insertions (*P* = 0.0005) and for deletions (*P* = 0.008). For all classes of variants, short indels, especially single-base pair indels, are the most abundant (Figure 5). Figure 5 depicts indels up to 20 bp, though observed variants include indels of up to 279 bp in length.

Observed short indels in FN lines include 80 located within exons, with 34 of these changes introducing frameshifts that alter the amino acid sequence of a transcript. Chromosomal locations and gene ontology for the 20 variants that introduced frameshifts in these 34 transcripts are available in Supplementary Table S1. Based on the VeP (McLaren *et al.* 2016) analysis of all variants in both datasets, 26% of all coding variants in FN lines induce a frameshift. This compares to 3.9% of rare standing coding variation and 2.8% of common standing coding variation. The much more frequent observation of the disruption of coding genes among variants in FN lines maintained under single seed descent reflects the limited opportunity for purifying selection to act on de novo mutations.

Single-base pair indels are more clearly identifiable as a single mutational event (Hahn 2018), and thus, the nature of the nucleotide change is readily identified. The mutations in FN lines and standing variants show a similar tendency toward deletions of A or T. Previous studies in soybean have reported observed GC content of 33% (Swaminathan *et al.* 2007), similar to the 34% observed here (Supplementary Figure S7A). Single-base pair insertions are even more heavily dominated by A and T changes (Supplementary Figure S7B).

## Discussion

We examined levels of nucleotide sequence diversity in 27 soybean lines subjected to FN mutagenesis. Most assays of genetic variants include variants arising from a mixture of mutagenic processes (Zhu *et al.* 2020). The primary difficulty is distinguishing FN-induced single-nucleotide variants from those that arise from spontaneous mutations. The nature of mutations, their rate of occurrence, and the nucleotide sequence context in which variants are observed can be used to assess the proportion of soybean variants that were caused by FN mutagenesis as opposed to spontaneous processes. The observed SNP variants in the FN lines are primarily C→T*, which is the most abundant class of variants in soybean generally and occur at a rate that is consistent with variants expected to arise spontaneously in the seed stock or generations of inbreeding following mutagenesis. An examination of *A.thaliana* lines treated with 60 Gy of FN irradiation identified a ∼50-fold single generation increase in mutation rate (Belfield *et al.* 2012). The same study noted an increase in G→T transversions that is not observed in our soybean lines. Aside from the difference in species examined, the highest level of FN exposure in the present study was 30 Gy, which may account for some of the differences in observed patterns of mutation. The lack of a distinctive pattern of mutation contrasts with results previously published on the effects of chemical mutagens. This includes sodium azide in barley, where A→G* transitions predominate (Olsen *et al.* 1993; Talamè *et al.* 2008) or ethyl methanesulfonate, which can result in the alkylation of guanine, creating primarily C→T* transitions (Henry *et al.* 2014).

The most distinctive aspect of FN mutagenesis found in the present study is the induction of many small deletions (Figure 5). This result closely aligns with the observations of Belfield *et al.* (2012), where the 253 single-base pair deletions were the most abundant class of indel variants. Among the 34 frameshift that will oftentimes alter gene function, 18 are single-base deletions. FN-induced indels include proportionally fewer short 1–3 bp insertions. The induction of small deletions that eliminate gene function is highly desirable for phenotypic screens aimed at identifying the genetic basis of observable phenotypes through gene knockdown or knockout. Of course, the primary challenge with FN mutagenesis is that double-stranded DNA breaks can create large structural variants, including deletions, which impact many genes, posing a challenge to associate phenotypes with individual mutations (Schneeberger 2014).

Efforts to identify induced nucleotide sequence changes must actively address several analytical and experimental issues. The relative magnitude of these issues, as measured by the number of variants they contribute, includes the following: First, experimental contamination through “off-type lines” or lines subject to unintended hybridization (or outcrossing; Henry *et al.* 2014) can contribute millions of variants. Second, for reference-based read mapping, the differences between the line being investigated and the reference genome must be identified and eliminated from comparisons (Michno and Stupar 2018). Third, levels of expected heterozygosity among induced variants must be considered, and sequencing depth must be sufficient for the identification of heterozygous variants. Most experimental designs in plants involve inbred mutagenized lines. The number of generations of inbreeding after mutagenesis must be tracked to identify variants and to accurately estimate expected heterozygosity at induced mutations (Henry *et al.* 2014). Fourth, heterogeneity in experimental lines (Haun *et al.* 2011; Stec *et al.* 2013) subject to the initial treatment can contribute large numbers of variants that continue to segregate or occur as fixed differences among treatment lines (Michno and Stupar 2018). Fifth, sequencing errors can contribute to low-quality variant calls (Li *et al.* 2008). The sources of these errors include base call errors, inadequate coverage, poor mapping quality (Figure 1), repetitive DNA sequences, paralogous loci where reads cannot map uniquely, and quality issues in a reference genome (Morrell *et al.* 2011).

From a biological perspective, we find that the SNP frequency and sequence context of variants in the FN lines are similar to expectations based on the spontaneous rate of mutation over the generations of the experiment. This finding suggests that a large portion of single-nucleotide variants in the FN lines arise as spontaneous mutations, rather than as a direct result of the FN mutagenic process. Meanwhile, we find that FN mutagenesis induces many short indels, a portion of which result in frameshift mutations within the coding portions of genes. FN mutagenesis has long been exploited for experiments seeking to knock down or knock out single genes and to eliminate dominant traits in breeding programs; short indel-induced frameshift mutations are likely contributors.

From a regulatory viewpoint, these findings form a baseline with which to evaluate the relevance and importance of other mutations that result from the application of biotechnology. Applications of targeted genetic modifications in plants are rapidly expanding (Modrzejewski *et al.* 2019). Although the quantification of off-target effects from various genome editing applications is confounded by differences in methodology, the numbers reported are low, perhaps to the point of reflecting the background mutation rate. In contrast, technologies long considered safe can be quite disruptive at the whole-genome level.

## Conclusions

The widespread use of targeted genetic modifications, particularly with CRISPR-based approaches, has reinvigorated a regulatory debate about “unintended” changes in edited crop genomes. To the extent that gene editing mimics naturally occurring DNA changes and those induced with mutagenesis, standing variation and variation in mutagenized crops provide a baseline for the number and types of mutations that have a history of safe use and therefore do not present increased safety risks. Among the 27 soybean lines resequenced in our study, the number of new mutations induced by FN radiation exposure is orders of magnitude lower than the number of differences observed between lines because of naturally occurring standing variation. The single-nucleotide variants in lines subject to FN exposure are very similar to naturally occurring variants, particularly the rarest natural variants that are likely to be most similar to new mutations. Of these, C-to-T transitions predominate in both standing variation and FN lines. The nucleotide sequence flanking variants are not markedly different in standing variation and FN-mutagenized lines. FN lines carry more short indels, particularly 1-bp deletions that disrupt coding genes. FN-treated lines carry an average of 85 mutations, including at least one loss-of-function mutation per accession, though none of the samples resequenced for this analysis show a visible phenotype.

## Data availability

The Valliyodan *et al.* (2016) resequencing dataset with 106 soybean samples is available from the NCBI Sequence Read Archive (SRA) accession code SRP062245. Previously published M92-220 resequencing datasets are at SRA code SRP036841. Newly collected resequencing data reported here are available at SRA code PRJNA670564. The processed datasets supporting the conclusions of this article are available in a DRUM archive, https://doi.org/10.13020/0s9b-p605 (Accessed: 2021 December 21). Scripts for data processing and figure generation are available in the Context Variants Soy repository, in https://github.com/MorrellLAB/Context_Variants_Soy (Accessed: 2021 December 21) and in the SNP_Context repository, in https://github.com/carte731/SNP_Context (Accessed: 2021 December 21).

Supplementary material is available at *G3* online.

## Supplementary Material

jkab431_Supplementary_Figure_S1

jkab431_Supplementary_Figure_S2

jkab431_Supplementary_Figure_S3

jkab431_Supplementary_Figure_S4

jkab431_Supplementary_Figure_S5

jkab431_Supplementary_Figure_S6

jkab431_Supplementary_Figure_S7

jkab431_Supplementary_Table_S1

jkab431_Supplemental_Material_Legends
