# Supplementary material for: Fast neutron mutagenesis in soybean enriches for small indels and creates frameshift mutations
Source: G3 (Bethesda). 2021 Dec 15;12(2):jkab431. doi: 10.1093/g3journal/jkab431 (PMC9335934; doi:10.1093/g3journal/jkab431)
Supplement: jkab431_Supplementary_Figure_S1 [file jkab431_supplementary_figure_s1.pdf]

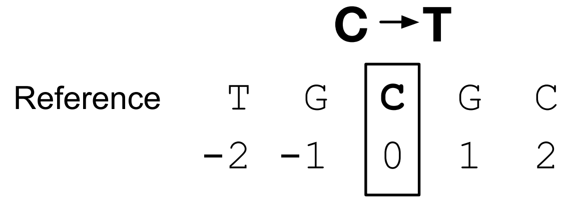
C → T
Reference
T G C G C
−2 −1 0 1 2
