# Supplementary figures and images for: Fast neutron mutagenesis in soybean enriches for small indels and creates frameshift mutations
Source: G3 (Bethesda). 2021 Dec 15;12(2):jkab431. doi: 10.1093/g3journal/jkab431 (PMC9335934; doi:10.1093/g3journal/jkab431)

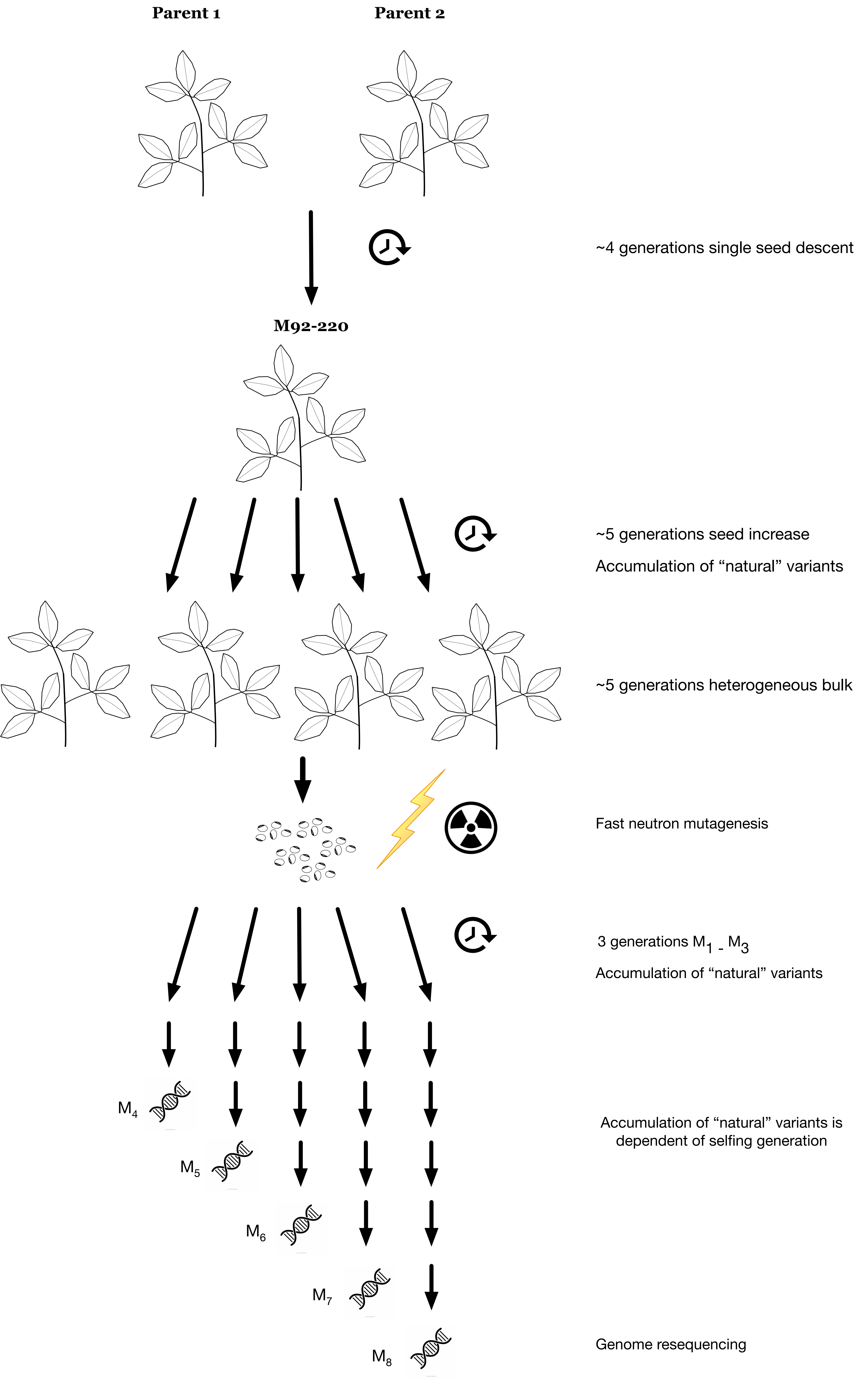

Supplement: jkab431_Supplementary_Figure_S2 [file jkab431_supplementary_figure_s2.pdf]

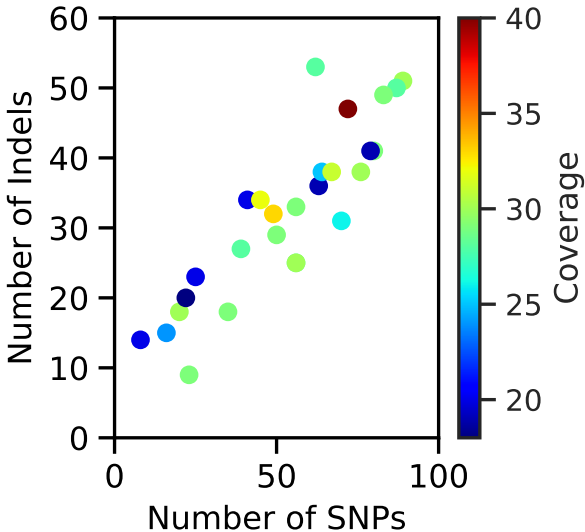

Supplement: jkab431_Supplementary_Figure_S3 [file jkab431_supplementary_figure_s3.pdf]

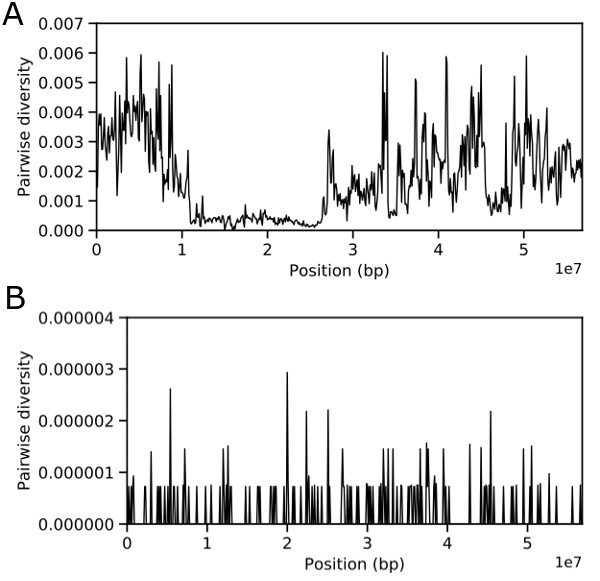

Supplement: jkab431_Supplementary_Figure_S5 [file jkab431_supplementary_figure_s5.jpeg]

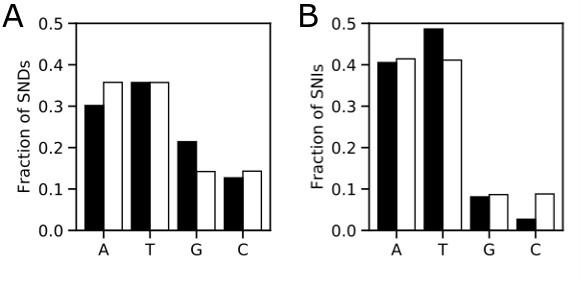

Supplement: jkab431_Supplementary_Figure_S7 [file jkab431_supplementary_figure_s7.jpeg]
