# Supplementary material for: Fast neutron mutagenesis in soybean enriches for small indels and creates frameshift mutations
Source: G3 (Bethesda). 2021 Dec 15;12(2):jkab431. doi: 10.1093/g3journal/jkab431 (PMC9335934; doi:10.1093/g3journal/jkab431)
Supplement: jkab431_Supplementary_Figure_S4 [file jkab431_supplementary_figure_s4.pdf]

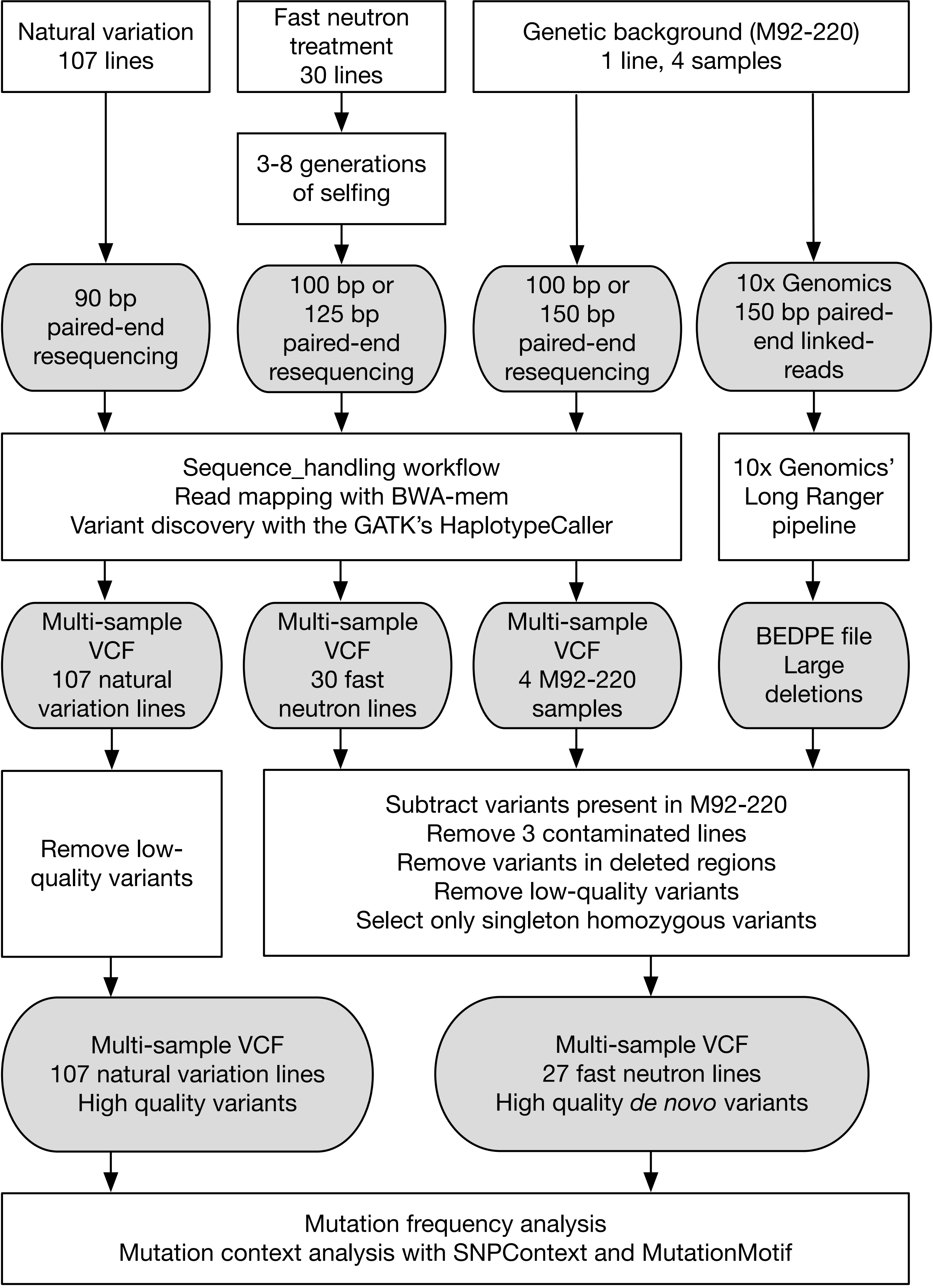

Figure 1: Data generation and a processing schematic for all samples in the present study is shown. Variants were called against the Williams 82 reference genome from the Illumina resequencing data using GATK best practices. SNPs cannot be accurately called in genomic regions where the mutagenized line = (M92-220) differs from reference by structural changes (e.g., deletions). Structural differences between M92-220 and Williams 82 have filtered out.
