# Supplementary material for: Fast neutron mutagenesis in soybean enriches for small indels and creates frameshift mutations
Source: G3 (Bethesda). 2021 Dec 15;12(2):jkab431. doi: 10.1093/g3journal/jkab431 (PMC9335934; doi:10.1093/g3journal/jkab431)
Supplement: jkab431_Supplementary_Figure_S6 [file jkab431_supplementary_figure_s6.pdf]

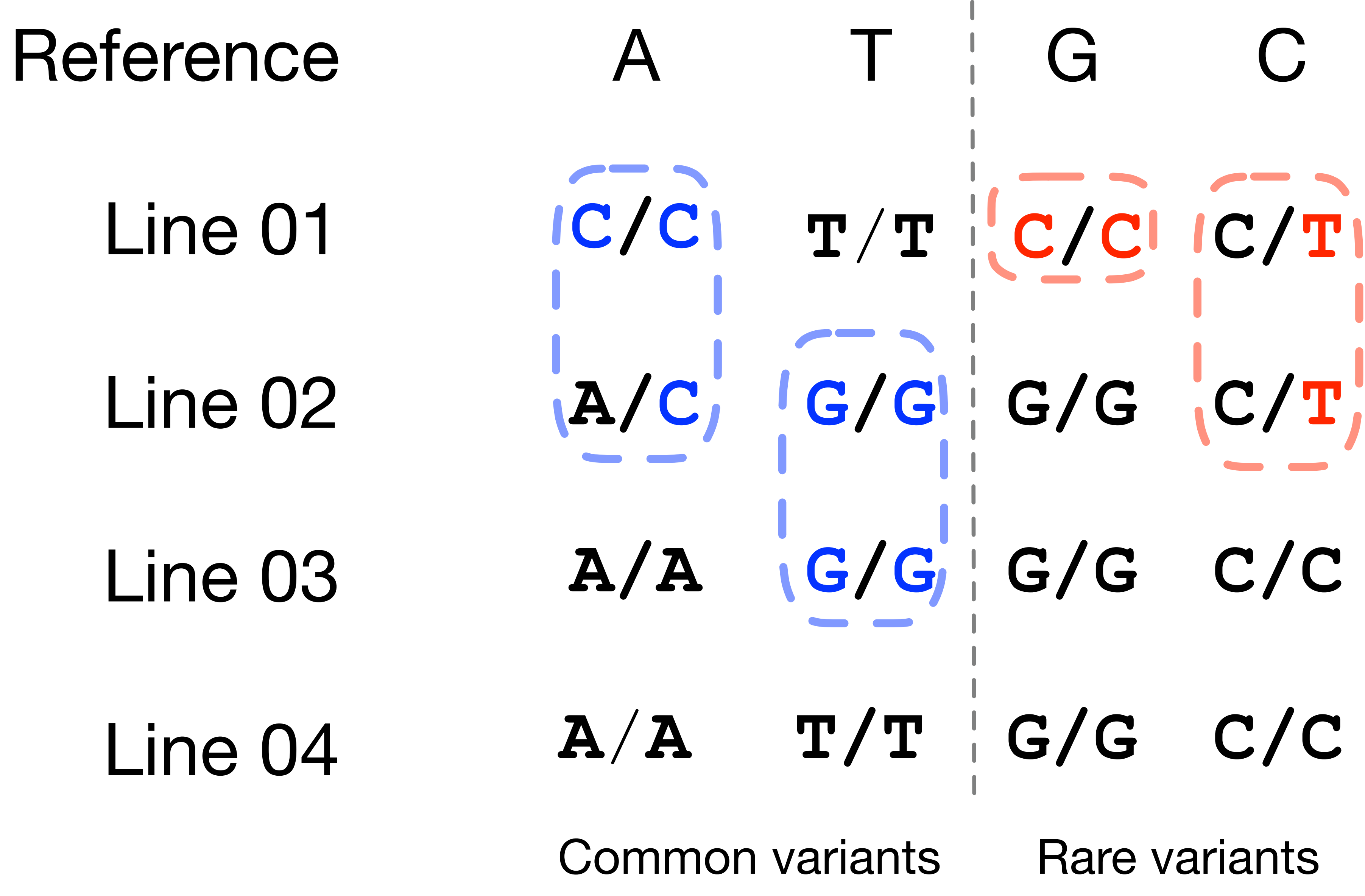

Reference
A
T
G
C
Line 01
C/C
T/T
C/C
C/T
Line 02
A/C
G/G
G/G
C/T
Line 03
A/A
G/G
G/G
C/C
Line 04
A/A
T/T
G/G
C/C
Common variants
Rare variants
