# Supplementary material for: Fast neutron mutagenesis in soybean enriches for small indels and creates frameshift mutations
Source: G3 (Bethesda). 2021 Dec 15;12(2):jkab431. doi: 10.1093/g3journal/jkab431 (PMC9335934; doi:10.1093/g3journal/jkab431)
Supplement: jkab431_Supplemental_Material_Legends [file jkab431_supplemental_material_legends.docx]

Supplemental tables and figures

**Table S1.** Chromosomal location, gene ontology, and additional information for the frameshift mutations discovered in the FN panel.

**Figure S1.** The sequence context for each variant involves the nucleotide sequence in the reference genome that immediately flank a variant nucleotide site. The variant site (in the example, a C->T change) is identified as the “0” position, and positions to the right of the variant identified by positive values and positions to the left identified as negative values.

**Figure S2.** The schematic depicts the creation of the fast neutron experimental population. The schematic is intended to depict all of the generations in which observed mutations could have arisen. Thus we begin with the cross that created the M92-220 line. Treatment lines vary in the number of generations of self-fertilization after mutagenesis.

**Figure S3.** The number of SNPs and number of indels in fast neutron treated lines plotted relative to average genome-wide resequencing coverage.

**Figure S4.** The schematic depicts the data generation and analysis process for all resequenced lines in the experiment. Variants were called against the Williams 82 reference genome from the Illumina resequencing data using GATK best practices. SNPs cannot be accurately called in genomic regions where the mutagenized line (M92-220) differs from the reference genome by structural changes (e.g., deletions). Structural differences between M92-220 and Williams 82 have been filtered out.

**Figure S5.** Pairwise diversity in standing variation (panel A) versus FN lines (panel B) across chromosome 1. Note the difference in scale for the y-axis.

**Figure S6.** Standing variation was partitioned into common and rare variants using the scheme depicted. Rare variants (shown in red) are found in two or fewer copies in the panel (either in the homozygous or heterozygous state), while common variants (shown in blue) occur three or more times.

**Figure S7.** Nucleotide composition of single nucleotide deletions (SNDs) (panel A) and single nucleotide insertions (SNIs) (panel B). Variants in FN lines are shown in black, while variants identified as standing variation are shown in white.
